# COVID-19 associated acute demyelination masquerading as stroke: a case report

**DOI:** 10.1186/s43055-021-00410-7

**Published:** 2021-01-20

**Authors:** Krati Khandelwal, Monika Puranik, Vivek Gupta, Gaurav Khandelwal, Pranav Kumar Dave, Makarand Hirve

**Affiliations:** 1 Department of Radiology, LN Medical College and JK Hospital, Bhopal, India; 2 Department of Cardiology, All India Institute of Medical Sciences, Bhopal, India; 3 Department of Neurology, LN Medical College and JK Hospital, Bhopal, India

**Keywords:** COVID-19, Symmetrical, Demyelination, Internal capsule, Stroke, MRI

## Abstract

**Background:**

During the recent outbreak of COVID-19, various atypical extrapulmonary manifestations are being seen, including neurological ones. Reported cases mainly include encephalopathy, myelitis, and cranial nerve involvement. This case describes uncommon neuroradiological finding in the context of COVID-19.

**Case presentation:**

We report an atypical case of COVID-19 presenting with stroke-like episode, with MRI brain showing isolated bilateral posterior internal capsule involvement. This has rarely been reported in literature.

**Conclusion:**

As the numbers of COVID-19 cases are increasing, such atypical presentations should be kept in mind.

## Background

COVID-19 pandemic continues to spread across the world with critical challenges for the public health and medical communities globally. Its manifestations are predominantly respiratory, with multiorgan dysfunction in severe cases. Neurological involvement is not common, but data is growing [1]. There have been reports of encephalopathy, myelitis, and cranial nerve involvement in people infected by COVID-19 [2–4]. This case describes isolated bilateral symmetrical demyelinating lesions presenting with stroke-like clinical presentation in the context of COVID-19.

## Case presentation

**Fig. 1:**
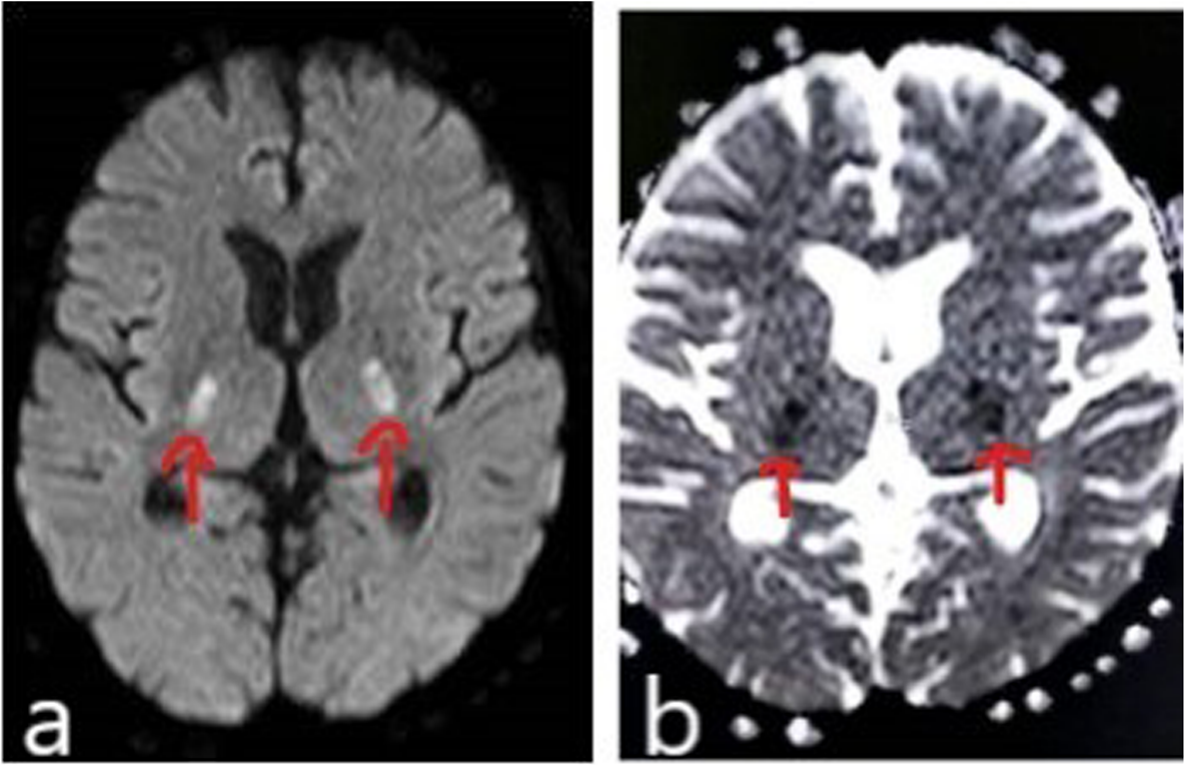
DWI (**a**) and corresponding ADC (**b**) images show restricted diffusion in posterior limbs of bilateral internal capsule (red arrows)

**Fig. 2:**
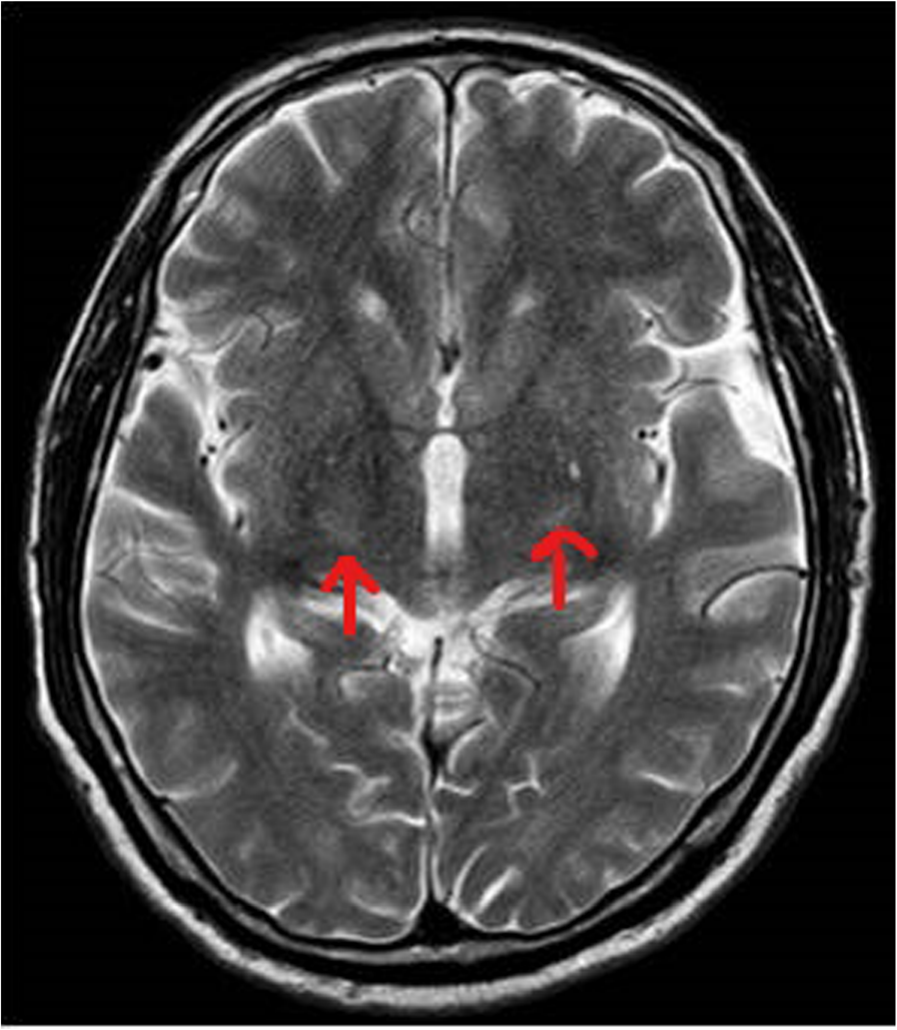
Axial T2 WI at the level of ganglio-thalamic region showing hyperintensity in posterior limbs of bilateral internal capsules (red arrows)

**Fig. 3:**
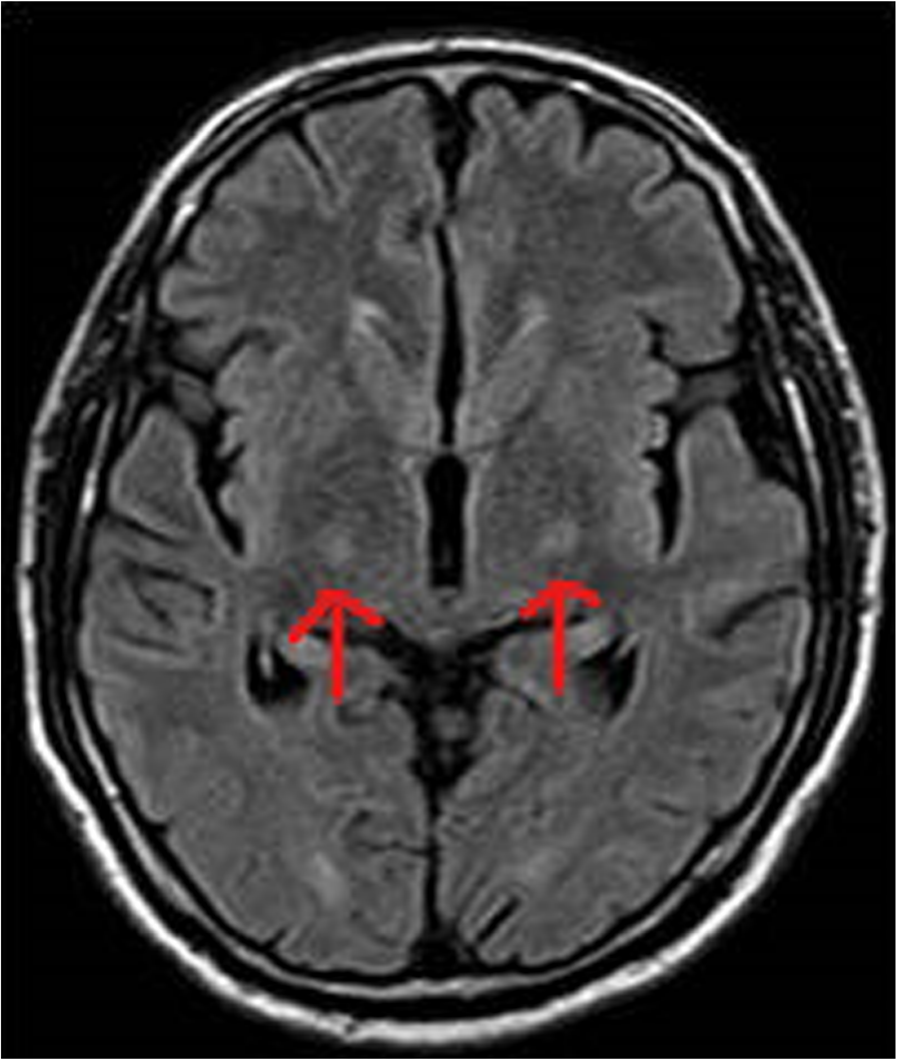
Axial FLAIR sequence at the level of ganglio-thalamic region showing hyperintensity in posterior limbs of bilateral internal capsules (red arrows)

**Fig. 4:**
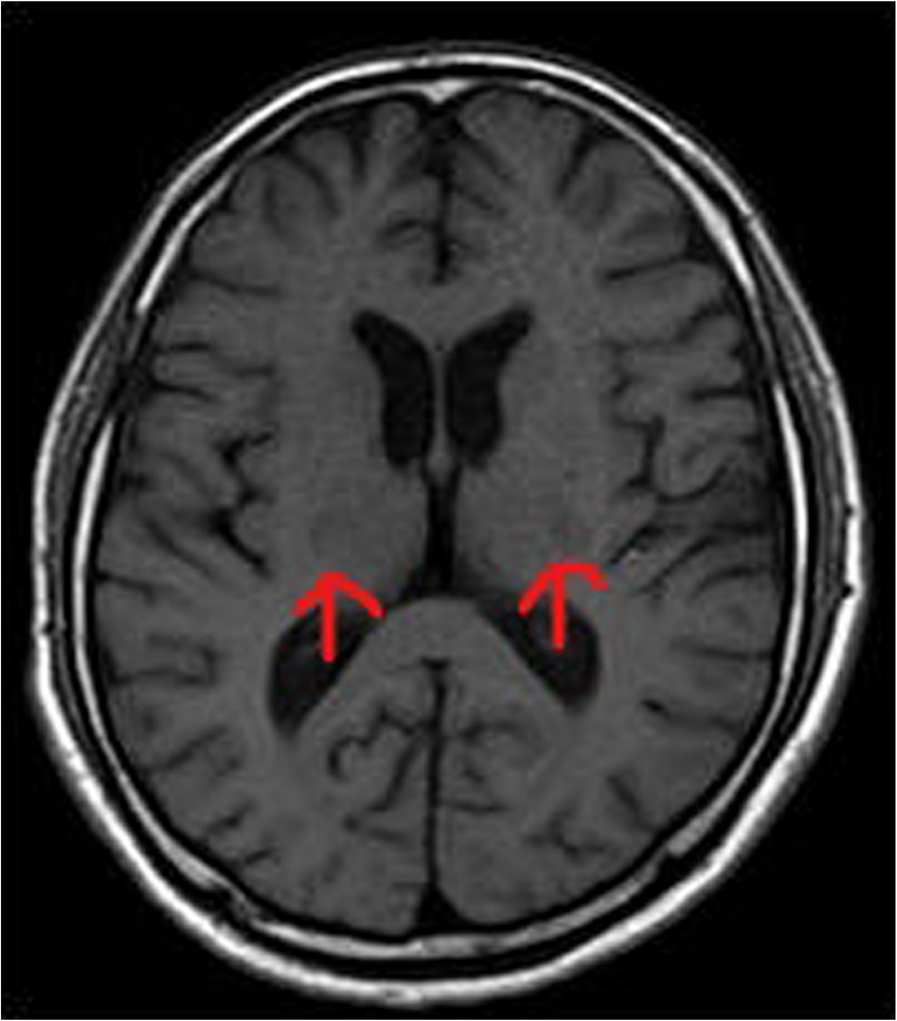
Axial T1 WI at the level of ganglio-capsular region showing hypointensity in posterior limbs of bilateral internal capsules (red arrows)

**Fig. 5:**
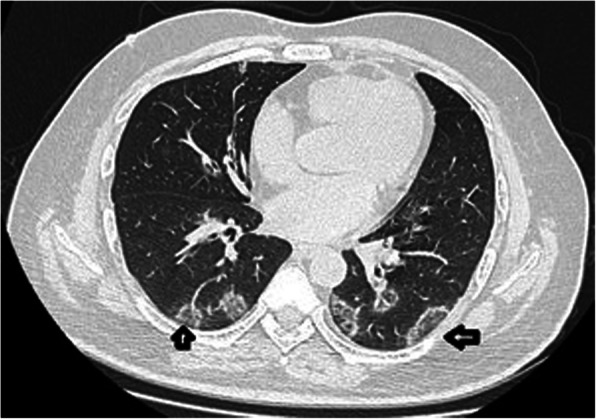
Axial HRCT chest image of the patient (120 KV 100 mAs bone algorithm) at the level of heart showing bilateral multifocal ground glass lesions (open arrows) in both lower lobes in peripheral distribution

A 58-year-old male, known hypertensive and diabetic, presented with sudden onset of slurring of speech for 1 day. He also had history of fever since 2 days, prior to these complaints. On examination, he had dysarthria, anarthria, and had difficulty in meaningful writing. However, he was conscious, alert, with no sensory or other motor weakness. Cranial nerve functions were intact. There were no meningeal signs or bladder and bowel dysfunction. Clinically, stroke was suspected, for which he was referred to the radiodiagnosis department for MRI brain. MRI showed bilateral symmetrical diffusion restriction on DWI (Fig. 1a, b) involving posterior limbs of both internal capsules. These involved areas that were hyperintense on T2W and FLAIR sequence (Figs. 2, 3) and were hypointense on T1W sequence (Fig. 4). No specific vascular territorial infarct was seen. In view of history of fever, RT PCR swab test for COVID-19 was sent, which subsequently showed positive result. HRCT chest was also done which showed bilateral multifocal peripheral ground glass opacities (Fig. 5) suggestive of COVID-19 pneumonia. The patient was given remdesivir, methylprednisolone, antibiotics, and other supportive treatment, at the discretion of the clinician. His neurological symptoms showed gradual mild improvement during the hospital stay.

## Discussion

Neurological involvement in COVID-19 was initially suggested by multiple case reports showing encephalitis/acute necrotizing encephalopathy in MRI of affected patients [2–4].

A recent multicenter study discussed neuroimaging findings in 37 patients of COVID-19 with neurological symptoms and abnormal MRI brain (excluding ischemic infarcts and cerebral venous thrombosis) [5]. The most frequent neuroimaging findings described in it were signal abnormalities located in the medial temporal lobe in 43% patients; non-confluent multifocal white matter hyperintense lesions on FLAIR and diffusion, with variable enhancement and associated with hemorrhagic lesions in 30% patients; and in 24% patients, extensive and isolated white matter micro hemorrhages were detected. Few cases of transverse myelitis associated with COVID-19 have also been reported [6, 7].

Various mechanisms have been described by which the central nervous system is affected. The human coronavirus (HCoV) has neuroinvasive capacities and may be neurovirulent by two main mechanisms: viral replication into glial or neuronal cells of the brain or autoimmune reaction with a misdirected host immune response [8, 9].

Kihira et al. in their case series of 5 patients also suggests strong association between hypoxia and leukoencephalopathy [10].

Symmetric confluent leukoencephalopathy favors a component of post-hypoxic leukoencephalopathy, and more focal white matter lesions (particularly if the spinal cord or the posterior fossa is involved) may suggest post viral autoimmune demyelination [10].

Demyelinating lesions following COVID-19 have rarely been reported. Zoghi et al. reported a case showing demyelination of bilateral posterior internal capsules extending to ventral pons and also with splenial involvement along with longitudinally extensive transverse myelitis of cervical and thoracic cord [11].

We report isolated symmetrical demyelination of bilateral posterior internal capsules, which showed restricted diffusion on DWI sequence, appearing hyperintense on T2/FLAIR sequences and hypointense on T1 sequence. There was sparing of the brain stem and spinal cord. The absence of grey matter involvement, and no hemorrhage or cavitation, suggests demyelination, rather than necrotizing encephalomyelitis. Although our patient had stroke-like presentation, no specific vascular territory was involved. Bilateral internal capsule involvement associated with COVID-19 infection and acute presentation suggested it to be COVID-19-associated demyelination. To the best of our knowledge, this is the first such reported case.

Imaging differentials to be considered are metabolic leukoencephalopathy, amyotrophic lateral sclerosis, and neuromyelitis optica syndromes. These can be ruled out on the basis of history and imaging findings.

## Conclusion

COVID-19 associated with demyelination, though mostly presents like acute disseminated encephalomyelitis, in rare cases can present like acute isolated symmetrical internal capsule demyelination. As the number of patients with COVID-19 increases worldwide, clinicians and radiologists should be aware of these findings and have high index of suspicion for COVID-19 in patients presenting with stroke-like episodes.

## Data Availability

Available

## Abbreviations

MRI: Magnetic resonance imaging
DWI: Diffusion-weighted imaging
RT PCR: Reverse transcriptase polymerase chain reaction
FLAIR: Fluid-attenuated inversion recovery
HRCT: High-resolution computed tomography
